# Immune enhancement and disease resistance against *Aeromonas hydrophila* infection by dietary *Lactobacillus plantarum*-fermented *Moringa oleifera* leaves in *Oreochromis niloticus*

**DOI:** 10.3389/fvets.2025.1557671

**Published:** 2025-04-25

**Authors:** Ghada S. Abdelkader, El-Sayed Y. El-Naenaeey, Hossam M. Abdallah, Ehsan H. Abu-Zeid, Ibrahim F. Rehan, František Zigo, Gamal A. Elmowalid

**Affiliations:** ^1^Department of Microbiology, Faculty of Veterinary Medicine, Zagazig University, Zagazig, Egypt; ^2^Department of Forensic Medicine and Toxicology, Faculty of Veterinary Medicine, Zagazig University, Zagazig, Egypt; ^3^Department of Husbandry and Development of Animal Wealth, Faculty of Veterinary Medicine, Menoufia University, Shebin Alkom, Egypt; ^4^Department of Pathobiochemistry, Faculty of Pharmacy, Meijo University, Nagoya, Japan; ^5^Department of Nutrition and Animal Husbandry, University of Veterinary Medicine and Pharmacy, Košice, Komenského, Slovakia

**Keywords:** *Lactobacillus plantarum*-fermentation, *Moringa oleifera*, Nile tilapia, innate immunity, phagocytosis, immune-related genes, HPLC phytochemical analysis

## Abstract

For enhancing the nutritional characteristics of *Moringa oleifera* leaves (MOLs), the present research set out to examine the effect of MOLs fermented by *Lactobacillus plantarum* (MOLF) or MOLs powder (MOLP) on innate immunity defense and resilience to *Aeromonas hydrophila* challenge in *Oreochromis niloticus*. A 30-day experiment was conducted with 180 Nile tilapia fingerlings, divided randomly into five equal-sized groups of 36 fingerlings, three replicates per group. The 1st control fish received a basal meal devoid of any supplements. The 2nd MOLP-L and the 3rd MOLP-H fish received basal meals enriched with low and high levels of MOLP (50 g or 100 g/kg diet). The 4th MOLF-L and 5th MOLF-H fish received basal meals enriched with low and high levels of MOLF (50 g or 100 g/kg diet). Ferulic acid, gallic acid, caffeic acid, and p-coumaric acid were the primary phenolic components identified by HPLC in the fermented MOLs. Meanwhile, naringenin, rutin, quercetin, kaempferol, luteolin, apigenin, and catechin were the main flavonoids detected. The results revealed that MOLF dietary supplementation enhanced the immune-related outcomes more significantly (*P* < 0.05) than MOLP in a dose-related manner. Supplementation of MOLF increased serum nitric oxide and lysozyme levels, phagocytic index, phagocytic %, hepatic superoxide dismutase, and glutathione, yet declined the levels of malondialdehyde more significantly (*P* < 0.05) than the MOLP. The proinflammatory genes *IL1*β, *TNF*α, and *IL-2* were significantly (*P* < 0.0.05) down-regulated. In contrast, the expression of the *IL-10* gene was markedly upregulated in the spleen and head kidney (anterior) post *A. hydrophila* challenge in the MOLF-groups than the MOLP-groups. MOLF-supplemented groups showed a significantly (*P* < 0.05) enhanced relative proportion of survivorship and survival rates but decreased the *A. hydrophila* bacterial load (CFU) compared to the MOLP-supplemented groups. In conclusion, our findings have offered new insights into the promising immune-enhancing outcome of MOLF as a dietary supplement for immune augmentation against disease challenges in Nile tilapia.

## Graphical Abstract

Nutritional supplement of *L. plantarum* fermented *M. oleifera* leaves to Nile tilapia feeds could enhance the immune-related responses, antioxidant potential, and mRNA regulation of immunity-related genes, along with enhancing Nile tilapia resistance to *A. hydrophila* challenge. 
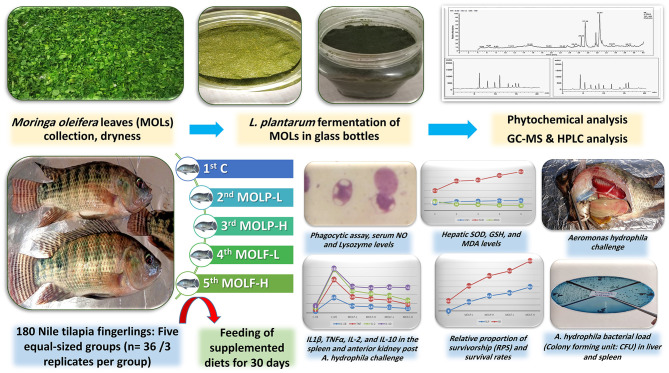

## 1 Introduction

Aquaculture delivers essential nourishment to the human population through high-quality proteins mainly sourced from several fish species (1). Aquatic species, especially fish, are extensively cultivated and significantly alleviate hunger in the human population (2). Nile tilapia (*Oreochromis niloticus*) constitutes the foundation of the aquaculture industry, yielding significant economic benefits owing to its superior flesh quality, competitive market pricing, elevated survival rate, and efficient feeding practices (3). The Nile tilapia's rapid growth rate, inexpensive production costs, and strong resistance to harsh circumstances make it one of the most significant commercial freshwater fish in aquaculture globally.

According to statistics from the U.S. Department of Agriculture's Foreign Agricultural Service, Egypt is the top producer of Tilapia globally and aquaculture in Africa (4). Outbreaks of bacterial infections, however, pose a significant danger to *O. niloticus* species and, by extension, Egypt's aquaculture industry, reducing fish production and killing an estimated 80% of the fish stock annually (5). *Aeromonas hydrophila* (*A. hydrophila*) is a Gram-negative, highly pathogenic bacterium that may accelerate epidemics and thrive in freshwater fish settings (6). It causes motile *Aeromonas septicemia* in cultured *O. niloticus*, which in turn causes bleeding septicemia and high mortality rates (3, 5).

The utilization of herbal remedies has shown a drastic increase in the 20^th^ century, as generally, all officially established medications are natural herbs (7). *Moringa oleifera* (*M. oleifera*), sometimes called the “Diamond of Plants”, “Tree of Life,” or “Tree of Wonders,” is a medical and dietary homologous herbal that has substantial health benefits for conditions like cancer, diabetes, hypertension, skin conditions, immunodeficiency, arthritis, and more (8, 9). Due to its capacity to enhance the healthiness, food conversion efficacy, performance during growth, and quality of products of various livestock animals, *M. oleifera* is extensively grown in nations of Asia and Africa and is preferred by the local population (10). According to research on aquatic animals, feeding MOL's extract may increase immune-related genes. Giant freshwater prawns (*Macrobranchium rosenbergii*) growth, immune-related gene expression, and resistance to *V. anguillarum* (11), while a 1% extract enhanced antioxidant activities and resistance to the *Photobacterium damselae* infection of whiteleg shrimp (*Penaeus vannamei*) (12). MOLP supplementation at 1.5 to 5% in the diet effectively improved immunity and reduced infection in Nile tilapia (*O niloticus*) (13). At 20% in the diet, it improved rainbow trout's (*Oncorhyncus mykiss*) immune parameters and antioxidant activity (14). Additionally, at 15% in the diet, it improved the growth and immune responses of Ctenopharyngodon idella (grass carp) (15). Common carp (*Cyprinus carpio*) showed enhanced immunity and stress tolerance when mixed with an aqueous extract at a rate of 20 milliliters per kilogram of feed (16). Moringa plant contains phytochemicals such as phenolic acid, flavonoids, carotenoids, alkaloids, tannin, lectin, and terpenoids (17), which have antioxidant, antistress, and immunostimulatory activities in gilthead seabream (*Sparus aurata*) and Nile tilapia (18–20).

Investigating *M. oleifera*'s main components and bioactivities and outlining its beneficial outcomes in nutritional treatments for animals and humans is extremely important and valuable (21). The miracle tree, or *M. oleifera*, is a member of the *Moringaceae* family widely cultivated worldwide because it remains the most nutrient-dense plant ever identified (22). MOLs are fully rich in vitamins C, B, A, E, and D; carotenes and beta-carotenes; minerals like iron and potassium; proteins; essential amino acids; fats; and numerous phytochemical components like alkaloids, terpenes, glucosinolates, flavonoids, phenols, and so on. It is a highly nutritious food with health issues-related significance (23).

*M. oleifera* has been dubbed a “superfood” and has increased in popularity. Almost every part of the tree is edible or has therapeutic qualities. Despite their nutrient-dense composition and many health advantages, MOLs are abundant in fundamental antinutritional inhibiting and toxic compounds, including alkaloids, tannins, oxalates, saponins, free phytates, and cyanogenetic glycosides. They have comparatively small concentrations of trypsin and amylase inhibitors. The listed antinutrients restrict the biological availability of vital nutrients and impede their digestion and absorption when ingested with nutrient-dense foods (24).

The enhanced usage of *M. oleifera* requires technical enhancement to guarantee the consumers' safety, improve the bioavailability of nutrients, and reduce production costs for health-promoting *M. oleifera* formulations (25). Various methods have been widely developed to minimize the impact of intrinsic toxicants and their secondary metabolites. These methods include membrane transformation, treatment with calcium chloride, radiation, exchange of ions, extraction of salty and aquatic contents, solubility differences, enzymatic handling, and microwave handling. Research indicates that food processing techniques such as drying, blanching, de-fatting, and sprouting retain the most significant impact on anti-nutritious agents and toxicants. These techniques improve the food's nutritional qualities and reduce or eliminate the activity of toxic substances through enzyme activity. Another method, heat and soaking treatments, destroyed proteins and resulted in water-soluble nutrients loss (26).

Fermentation is one of several processing methods that have been proposed as a method to improve plant foods' sensory qualities and nutrient delivery capabilities that are degraded by other processing methods (27). The anaerobic degradation of organic molecules by microorganisms and products of microbial origin is known as lactic acid fermentation. Due to this procedure; fermented foods have better texture, taste, and nutritional components (28). Many species of lactic acid bacteria (LAB), such as *L. plantarum, L. paracasei*, and *L. bulgaricus*, are known to carry out fermentation. These bacteria have probiotic qualities in addition to their capacity for fermentation (29). Moreover, due to their unique metabolic activity, LABs have antibacterial components. Proteolytic enzymes, bacteriocin synthesis, citrate absorption, metal ion resistance, bacteriophage resistance, polysaccharide biosynthesis, and antibiotics are a few of these (30).

Additionally, *Lactobacillus* species can degrade a variety of substances originating from plants, including phytates and polyphenols (31), trypsin-inhibiting agents, tannins (32), and oxalates (33). Few studies have documented the dynamic alterations in nutritional and botanical components during fermentation. In contrast, fewer studies have shown the antioxidative capabilities of *M. oleifera's* food-grade portions throughout the fermentation process.

Nowadays, microbial fermentation is used extensively as one of the fundamental methods for releasing nutrients. This is owing to catabolism throughout the fermentation, which breaks down complex molecules into simpler forms that cells can absorb readily via microbial action. For instance, carbohydrates are converted to glucose, proteins break down into amino acids, and lipids into fatty acids and glycerol (34). Furthermore, fermented substances' biological qualities are enhanced, and novel characteristics are often found (35). The health-harming antinutrients, such as oxalic and phytic acids, are broken down during fermentation. Fermentation can convert a plant's phenolic substances, thereby increasing the bioactivities of such components (36). Several enzymes produced by probiotics during the fermentation accelerate the hydrolysis of glycosidic bonds in some phenolic complexes. The total of free polyphenols could be enhanced by enzymatic hydrolysis (37).

Therefore, the purpose of this study was to investigate the effects of consuming fermented *M. oleifera* leaves using *L. plantaurum* or dried *M. oleifera* leaves powder on phagocytosis, innate immune status, oxidative stress, and immunity-linked gene transcriptional regulations in Nile tilapia, as well as disease resistance against *A. hydrophila* challenge.

## 2 Materials and methods

### 2.1 Collecting, authenticating, and fermenting MOLs

The Egyptian Scientific Society of Moringa, housed at the National Research Center in Dokki, Egypt, was contacted to get MOLs. The leaves were thoroughly rinsed under a stream of water to eliminate any sand or other contaminants. Subsequently, they were left to dry naturally in the open air without exposure to direct sunlight and were regularly rotated to prevent the growth of fungi. After 5 days, a fine powder was formulated by grinding MOLs, which could pass through a screen with a mesh size of 0.15 mm. The leaf meal was securely enclosed in polythene plastic bags, sealed, and stored at ambient temperature until needed.

Glass bottles measuring 1,000 mL were used. About 200 g of powdered MOLs were introduced into the bottles and then processed for 20 min under a sterile environment at 121°C. Afterward, the powder was cooled, and 10^8^ CFU/g of probiotic stock *L. plantarum* was inoculated into the powder. The medium's water content was based on the powders' absorption capacity. The mixture was homogenized in a sterile condition using a sterile glass rod. For 5 days, the fermentation process was done at 37°C (38).

### 2.2 Gas chromatography-mass spectrometry (GC-MS) analysis of fermented *M. oleifera* leaves

According to the protocol described by (39), a Trace GC-TSQ mass spectrometer (Thermo Scientific, Austin, TX, USA) equipped with a direct capillary column TG-5MS (0.25 mm × 30 m × 0.25 μm film thickness), the key compositions of the MOLF fine powder were examined. The column oven temperature was initially held at 50°C and then increased by the rate of 5°C/min to 250°C held for 2 min, then increased to the final temperature of 300°C by 30°C/min and held for 2 mins. The temperature in the MS transfer line and injector was maintained at 260°C and 270°C, respectively. Helium was employed as the carrier gas, and the flow rate was maintained at 1 mL/min. A 1 μL diluted sample was mechanically inoculated with 4 mins delayed solvent using a split-method Autosampler AS1300 paired with GC. In the full scan mode, electron ionization mass spectra were obtained spanning the m/z 50–650 range using an ionization voltage of 70 eV, covering. Around 200°C was used to heat the ion source. The GC components were identified by matching their mass spectra with those found in the WILEY 09 and NIST 14 databases (https://www.sisweb.com/software/ms/wiley.htm).

### 2.3 High-performance liquid chromatography (HPLC) quantitative analysis of the fermented *M. Oleifera* leaves extract of polyphenols

The polyphenols were measured according to the protocol described by (40, 41). Using HPLC equipment (Agilent 1100 series, USA) with an auto-sampling injector, solvent degasser, two LC-pump (series 1100), ChemStation software, and a UV/Vis detector (set at 250 nm for phenolic acids and 360 nm for flavonoids), we were able to analyze these chemicals. The particle size was 4.60 mm ^*^ 250 mm i.d., five μm, and the column used was an Eclipse C18. Two solvents were used in a gradient mobile phase to extract the phenolic components. Solvent A was methanol, while solvent B was a 1:2 acetic acid and water mixture. During the initial 3 mins of the gradient procedure, the concentration was maintained at 100% B. Following this, 50% eluent A was used for 5 mins. The concentration of A was raised to 80% for the next 2 mins and then decreased to 50% for the next 5 min. The wavelength for detection is 250 nm. The mobile phase was A, acetonitrile, and B, 0.2% (v/v) formic acid in the water, was employed for the flavonoid isolation using an isocratic elution (70:30) method. The separation was carried out with a solvent rate flow of 1 mL per min at 25°C five μL was the volume of every injection.

### 2.4 Ethical approval

Following the guidelines set out by the National Institutes of Health, the Zagazig University EAURC (Ethics of Animal Use in Research Committee) approved the experimental protocol, approval number ZU-IACUC/2/F/80/2024.

### 2.5 Fish management condition

A total number of 180 Nile tilapia fingerlings obtained from the Fish Research Center, Zagazig University, were utilized in the current investigation. The fish had a mean weight of 30 ± 3 g. This experiment was performed at Zagazig University's Faculty of Veterinary Medicine in the Fish Diseases and Management Department. The fish were grown up and acclimatized to the lab environment for 14 days. Tilapia was maintained in aquariums made of glass (30 × 40 × 80 × cm) containing 75 L of dechlorinated water. The laboratory photoperiod was at 12 h of light and 12 h of night. Daily at 8:00 am, following the elimination of accumulated fish excreta and cleaning, water was partially substituted for each tank with clean, dechlorinated water. Throughout the study period, the water-related parameters were followed those needed by tilapia for development (42). The water quality kept up at (a temperature of 26.50 ± 0.37 °C, pH of 7.67 ± 0.38, total ammonia nitrogen of 0.11 ± 0.01 mg/L, and dissolved oxygen of 6.80 ± 0.31 mg/L).

### 2.6 Experimental diets preparation and experimental protocol

Isonitrogenous and isocaloric basal diets were formulated according to the requirements outlined in the Nutrient Requirements of Fish (43). Formulated experimental diets were prepared to meet ' 'fish's nutritional requirements as shown in Table 1. The chemical components and nutritional needs of each basal diet were formulated according to the guidelines (44). The macro-Kjeldahl technique, petroleum ether, and a muffle furnace were employed to determine the crude proteins, crude lipids, and total ash contents of the prepared fish meals utilized in this study. The duration of the experiment spanned 30 days. Throughout the experiment, every day at 9:00 am, 12:00 pm, and 4:00 pm, the fish were given meals that were equal to 3% of their body weight according to the protocol (45). After the fish were weighed, they were randomly divided into five groups for the experiment. With 10 fish in each replication and 3 replicates totaling 30 fish, the experiment was conducted in triplicate. The 1st control set received basal meals devoid of supplements. The 2nd and 3rd sets received a basal meal supplemented with MOLP at 50 g and 100 g/kg diet, respectively, for 30 days. The 4th and 5th sets received basal meals supplemented with MOLF at 50 g and 100 g/kg diet for 30 days. For each diet, ingredients were thoroughly mixed, and then for each kg of diet, 100 mL of water was added. Using a standard kitchen blender, the last formulated paste of each diet was mixed. Then diets were made into pellets with the lab pellet instrument and allowed to dry for 1 day before storage in hygienic, sterile plastic containers at 4°C till usage.

**Table 1:** Formulation and chemical composition of the experimental diets.

**Ingredients**	**Experimental diets**				
	**Control item**	**MOLP-L**	**MOLP-H**	**MOLF-L**	**MOLF-H**
Fish meal	110	110	110	110	110
Yellow corn flour	330	330	300	330	300
Soybean meal 44%	290	290	290	290	290
Corn gluten meal 60%	120	120	120	120	120
Wheat bran	110	60	40	60	40
MOLP	0	50	100	0	0
MOLF	0	0	0	50	100
Premix-Mina 10^a^	10	10	10	10	10
Premix-Vitb 10^b^	10	10	10	10	10
Corn oil	20	20	20	20	20
Total	1,000	1,000	1,000	1,000	1, 000
**Chemical composition**					
Crude protein	303.5	303.9	305.2	304.3	305.9
Crude lipid	70.5	71.25	71.03	71.08	71.11
Crude fiber	38.1	40.1	42.9	42.3	41.5
Ash	67.2	66.7	68.3	68.8	69.2
Nitrogen free extract^c^	520.7	518.05	512.57	513.52	512.29
Gross energy (MJ/kg)^d^	18.92	18.91	18.84	18.84	18.85

^a^Composition of mineral premix kg^−1^: manganese, 53 g; zinc, 40 g; iron, 20 g; copper, 2.7 g; iodine, 0.34 g; selenium, 70 mg; cobalt, 70 mg, and calcium carbonate as carrier up to 1 kg. ^b^Composition of vitamin premix kg^−1^: vitamin A, 8,000,000 IU; vitamin D3, 2,000,000 IU; vitamin E, 7,000 mg; vitamin K3, 1,500 mg; vitamin B1, 700 mg; vitamin B2, 3,500 mg; vitamin B6, 1,000 mg; vitamin B12, 7 mg; biotin, 50 mg; folic acid, 700 mg; nicotinic, 20,000 mg; pantothenic acid, 7,000 mg. ^c^Nitrogen free extract = 1,000 – (crude protein + crude lipids + ash + crude fiber). ^d^Gross energy (GE) was calculated as 23.64, 39.54, and 17.20 kJ/g for protein, lipid, and NFE, respectively (43).

### 2.7 Sampling

Following the 30-day experimental study, six fishes were selected at random from every set. Then, blood was collected from every group in a sterile manner from the caudal vein in clean and dry tubes containing heparin for evaluation of the phagocytosis assay. Another blood sample was taken in clean tubes without anticoagulant, and then for 15 min, blood was centrifuged at 3,599 × g rpm for sera separations. Then collected serum samples were maintained at−20°C and used for the investigation of the innate immunity responses. Spleen and anterior kidney specimens were dissected from 3 fish/replicates from all experimental tilapia groups on the 5th days post *A. hydrophila* challenge and immediately kept in TRIzol reagent at −80°C till the molecular investigation. Liver tissue samples were obtained, isolated, rinsed with physiological saline, and subsequently homogenized and centrifuged at 4°C for 15 min at 664 × g. The resulting supernatants were then utilized to estimate oxidative stress-linked markers.

### 2.8 Phagocytosis assay

Phagocytic activity was done according to the designated routine (46, 47), with particular adjustments. In a sterile plastic tube, after adding one mL of the heat-inactivated *C. glabrata* to one mL of the calibrated live leukocyte solutions (leukocytes in RPMI 1640 and 5% of collected Tilapia sera). Then the tubes were kept in the incubator humidified with CO_2_ 5% and incubated for 30 min at 27°C. After centrifuging the tubes for 5 min at 2,500 rpm, the supernatants were collected using a Pasteur pipette, leaving a tiny portion used to resuspend the sediment. Leishman's stain was applied after slide smears were made from the deposit and allowed to air dry.

Phagocytic cells in 10 microscopic fields were counted randomly using the oil immersion lens under a light microscope. The number of phagocytic cells engulfing yeast cells and the number of yeast cells inside each phagocytic cell was counted. The following equations were applied to measure the phagocytic activity. Phagocytic percentage (Ph %) = (count of phagocytes engulfing yeast cells/total count of phagocytic cells) × 100. Phagocytic index (PhI) = (total count of engulfed yeast/count of phagocytic cells engulfing yeast cells) ×100.

### 2.9 Quantification of serum and hepatic biochemical indicators

The colorimetric determination method was used to measure the superoxide dismutase (SOD) enzyme, reduced glutathione (GSH), and malondialdehyde (MDA) using commercial diagnostic colorimetric kits provided by Bio-diagnostic Co. LTD, Egypt, with the following catalog No. SD 25 21, GR 25 11, and MD 25 29, correspondingly.

### 2.10 *A. hydrophila* challenge test

At the Fish Diseases and Management Laboratory, Faculty of Veterinary Medicine, Zagazig University, moribund fish were used to cultivate *A. hydrophila*. Liver and spleen tissue samples were collected under strict sterile conditions. After spreading inoculums onto plates of tryptic soya agar (TSA; Difco, Detroit, MI), plates were maintained at 37°C for 1 day. Next, from each plate, one colony was selected to be cultivated in novel fresh plates of Rimler-Shotts agar media (RS; Difco, Detroit, MI). Following the ' 'manufacturer's instructions, the isolates were classified using the standard examination of their morphological characteristics, biochemical responses, an API 20NE test kit (bioM'erieux, Marcy l′Etoile, France), and the VITEK^®^ C15 automated system for identifying bacteria (BioMerieux Inc., France). Fifteen fish from each set were inoculated through the peritoneum with 0.2 mL of virulent *A. hydrophila* (24-h-old culture, 1 × 10^8^ CFU) once the 30-day experimental period had ended. The selected dose for *A. hydrophila* is equivalent to the median lethal dose (LD_50_) established (48) for the same bacterial species. The LD_50_ was 2.8 × 10^8^ CFU/mL. A sub-lethal dose was used in the *A. hydrophila* challenge test. The challenged fish were examined every day for the following 14 days to report any behavior alterations, clinical observations, and mortalities. The mean mortalities in all fish groups were estimated to determine the relative percent survival (RPS) (49). The clinically ill and recently deceased fish were used to re-isolate and confirm *A. hydrophila* employing biochemical characters, gram stain, and colony characterization. The bacterial “isolates” chemical properties were examined with the VITEK^®^ compact (BioM'erieux).

### 2.11 qRT-PCR of immunity-linked genes in spleen and anterior kidney

The transcriptional levels of immune-linked genes *il1*β*, TNF-*α*, il-2*, and *il-10* were investigated in the spleen and anterior kidney of Nile tilapia. The total RNA was extracted by the use of the QIAamp RNeasy Mini kit (Cat. No. 51304; Qiagen, Hilden, Germany). The levels of RNA were determined at 260 nm using a Spectrostar NanoDrop TM 2000 spectrophotometer (Cat. No. ND-2000; Thermo Fisher, Santa Clara, CA, USA). The quality of the RNA was also assessed using this spectrophotometer. The Stratagene MX3005P RT-PCR equipment (Cat. No. PF1457N; Thermo Fisher, Santa Clara, CA, USA) performed using a single-stage RT-qPCR replication in triplets. This was done using a Quanti Tect SYBR Green RT-PCR Kit (Cat. No. 204243; Qiagen, Hilden, Germany). Melting curve monitoring was used to validate the PCR amplifications. The gene responsible for housekeeping, specifically glyceraldehyde-3-phosphate dehydrogenase (Gapdh), was used as an in-house guide to standardize the levels of regulation for the transcripts. The primer sequences used in RT-qPCR tests are listed in Table 2. The 2^ΔΔ*CT*^ technique was employed to evaluate the proportional mRNA regulation results of the investigated genes (50).

**Table 2:** Primers sequences, accession number, and product size for the quantitative RT-PCR for the analyzed genes in the spleen and anterior kidney tissues.

**Target gene**	**Forward primer**	**Reverse primer**	**bp**	**Accession no**.
*IL-2*	CATTAGCAGAGCGCAAGCAG	ACCCTGGTGCTTTGGTGAAA	122	XM_003445827.5
*IL-1β*	TGAAGCTTCTGTAGCGTGGG	CTCATGTCTGTCCGCTACCC	103	KF747686.1
*TNF-α*	TAGCTGGTTGGTTTCCGTCC	CAGGATCTGGCGCTACTCAG	184	NM_001279533.1
*IL-10*	TGAGCTTCTTGAGCCTGACG	CAGCATTTCTGTGGACCAGC	200	KP645180.1
*Gapdh*	GCAGACACTTCACCACGGTA	GGCCATCAATGACCCCTTCA	93	NM_001279552.1

IL1β, interleukin 1β; TNFα, tumor necrosis factor α; IL-2, interleukin 2; IL-10, interleukin 10; GAPDH, glyceraldehyde-3-phosphate dehydrogenase.

### 2.12 Statistical analysis

A version of IBM SPSS Statistics, version 21 (IBM; Armonk, New York, USA), was used to analyze the data obtained. One-way analysis of variance (ANOVA) was performed to determine the statistical differences within groups. Subsequently, Tukey's multiple range *post hoc* test was applied for comparisons between pairs. The mean values ± standard error (SE) were used to represent the data for each group. Significance was considered at a threshold of *P* < 0.05. The clinical signs and autopsy lesions were estimated by semi-quantitative methods: “0 = absence of lesion, 1 = mild, 2 = moderate, and 3 = severe alterations”. The Kaplan-Meier model was performed to determine the specific survival probability. Mortalities were assigned as 1 and survivors as 0, detected every 24 h. Variations between the curves of MOLP or MOLF-exposed groups were tested for significance using the Log-rank Test, with a significance level of *P* < 0.05. Data visualization was also done using GraphPad Prism version 8 (GraphPad Software, San Diego, CA, USA).

## 3 Results

### 3.1 GC-MS investigation of fermented MOLs

**Figure 1:**
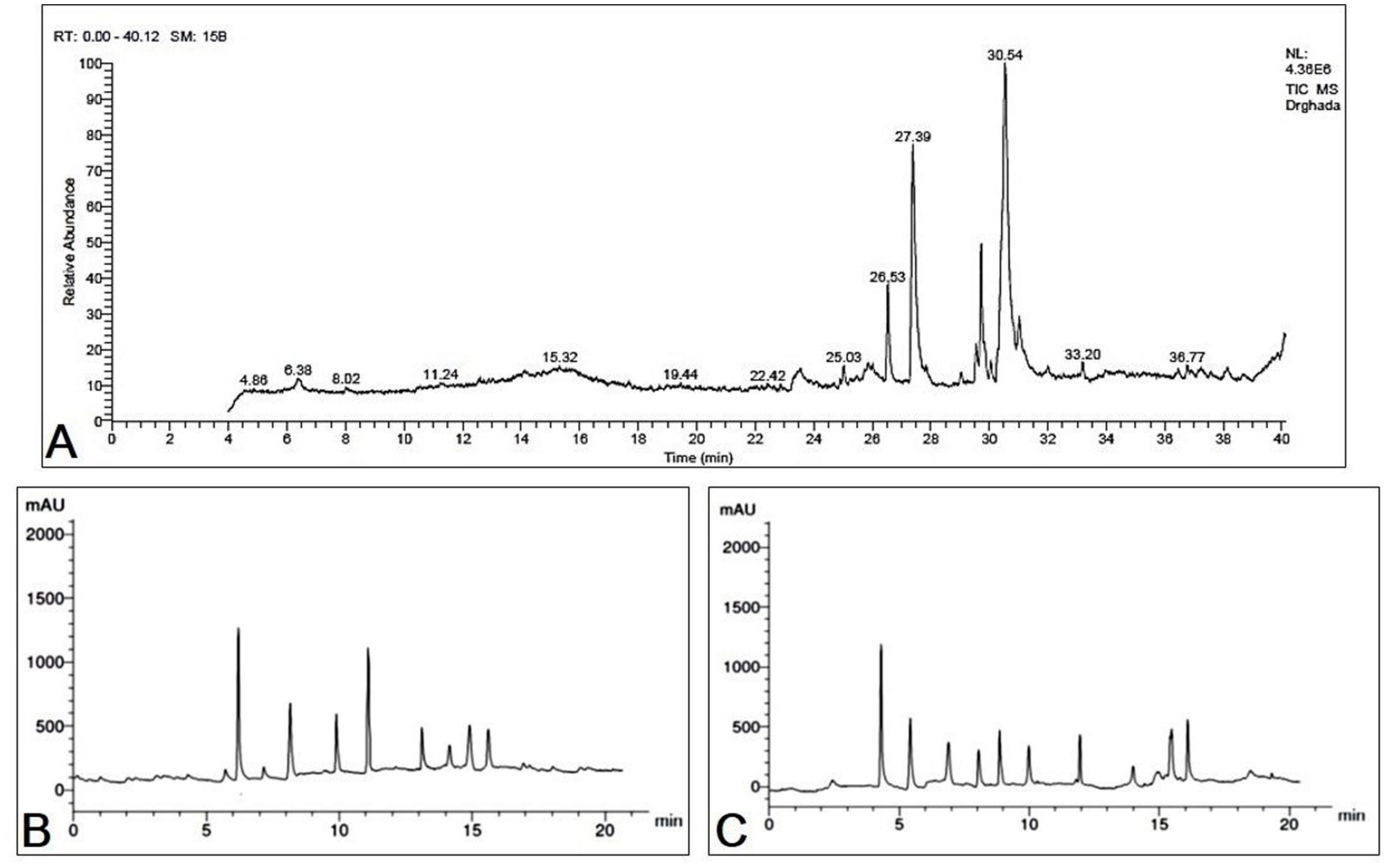
**(A)** GC-MS chromatogram represents the separated bioactive constituents of fermented *Moringa oleifera* leaves. HPLC chromatogram represents the separated bioactive constituents of fermented *Moringa oleifera* leaves ethanolic extract used in the present study. **(B)** Phenolic compounds, **(C)** flavonoids.

The GC-MS evaluation employed the screening of phytochemical contents. The compounds of MOLF, their respective durations of retention, and the proportion of the overall peak area that they constitute are presented in Table 3. Figure 1A displays the GC chromatogram that the GC-MS produced. In all, 33 signals have been determined. The principal components (which Area % is more than 1) of the fermented MOLs were (9E,12E)-9,12-(32.70%); octadecadienoyl chloride hexadecanoic acid (21.39%); methyl 9-octadecenoate (9.28%); 8,11,14-eicosatrienoic acid,(Z,Z,Z)- (3.02%); cyclopropaneoctanoic acid, 2-[[2-[(2-ethylcyclopropyl)methyl] cyclopropyl]methyl]-, methyl ester (2.42%); aspidospermidin-17-OL,1-acetyl-16-methoxy- (1.45%); 2-hexadecen-1-OL,3,7,11,15 tetramethyl-,[R-[R^*^,R^*^ (E)]]- (1.89%); 9-octadecenoic acid (Z)-,methyl ester (1.41%); panaxjapyne A (1.36%); 9,12,15-octadecatrienoic acid,2-(acetyloxy)-1 [(acetyloxy) methyl] ethyl ester, (Z,Z,Z)- (1.31%); 1-dodecaNol,3,7,11-trimethyl- (1.31%); 2H-oxecin-2-one,3,4,7,8,9,10- hexahydro-4-hydroxy-10-methyl-,[4S-(4R^*^, 5E,10S^*^)]-(1.25%); octadecanoic acid,9,10-epoxy-18-(trimethylsiloxy)-, methyl ester, CIS- (1.24%); 9-octadecenoic acid (Z)- (1.10%); Arachidonic amide, *N*-[5-hydroxy-n-pentyl]- (1.07%); and (9E,12E)-9,12-octadecadienoyl chloride (1.02%).

**Table 3:** List of phytochemical components of fermented *Moringa oleifera* leaves used in the current study and identified by GC-MS analysis.

**Peak no**.	**RT (min)**	**Peak area %**	**Compound name**	**MF**	**Molecular weight**	**Molecular formula**	**Peak area**
1	6.39	0.94	3-trifluoroacetoxydodecane	718	282	C14H25F3O2	699,917.08
2	6.46	0.52	Ethanimidothioic acid, 2-(dimethylamino)-N-[[(methylamino)carbonyl]oxy]-2-OXO-, methyl ester	676	219	C7H13N3O3S	387,330.13
3	22.86	0.59	2,5-octadecadiynoic acid, methyl ester	699	290	C19H30O2	435,665.10
4	23.34	1.31	1-dodecanol,3,7,11-trimethyl-	674	228	C15H32O	973,527.16
5	23.42	0.66	L-valine,N-[2-(chloroimino)-3-methy L-1oxobutyl]-	674	248	C10H17ClN2O3	489,012.68
6	23.46	0.75	7-methyl-Z-tetradecen-1-OL acetate	683	268	C17H32O2	560,288.96
7	23.54	1.25	2H-oxecin-2-one,3,4,7,8,9,10-hexahydro-4-hydroxy-10-methyl-,[4S-(4R^*^,5E,10S^*^)]-	676	184	C10H16O3	933,264.94
8	24.91	0.39	1-(([(butylamino)carbonyl] amino)sulfonyl)-4-methylbenzene	676	270	C12H18N2O3S	287,355.44
9	25.03	1.45	Aspidospermidin-17-OL,1-acetyl-16-methoxy-	795	370	C22H30N2O3	1,079,446.00
10	25.73	0.61	Aspidospermidin-17-OL,1-acetyl-16-methoxy-	795	370	C22H30N2O3	453,288.71
11	25.87	1.02	(9E,12E)-9,12-octadecadienoyl chloride	708	298	C18H31ClO	757,671.75
12	26.01	0.44	Undec-3-Ene 0.44 778 C11H22	778	154	C11H22	324,846.51
13	26.53	6.96	Hexadecanoic acid, methyl ester	855	270	C17H34O2	5,174,539.68
14	27.38	21.39	Hexadecanoic acid	852	256	C16H32O2	15,914,153.79
15	27.85	0.58	2-aminoethanethiolhydrogen sulfate (ester)	797	157	C2H7NO3S2	431,233.31
16	29.03	1.24	Octadecanoic acid,9,10-epoxy-18-(trimethylsilo xy)-, methyl ester, CIS-	683	400	C22H44O4Si	919,332.62
17	29.55	2.42	Cyclopropaneoctanoic acid, 2-[[2-[(2-ethylcyclopropyl)methyl]cyclopropyl]methyl]-, methyl ester	809	334	C22H38O2	1,802,406.31
18	29.72	9.28	Methyl 9-octadecenoate	909	296	C19H36O2	6,899,700.55
19	29.83	1.41	9-octadecenoic acid (Z)-,methyl ester	849	296	C19H36O2	1,052,472.89
20	30.05	1.89	2-hexadecen-1-OL,3,7,11,15-tetramethyl-,[R-[R^*^,R^*^ (E)]]-	826	296	C20H40O	1,407,967.03
21	30.28	0.68	13,16-octadecadiynoic acid, methylester	820	290	C19H30O2	504,451.76
22	30.53	32.70	(9E,12E)-9,12-octadecadienoyl chloride #	857	298	C18H31ClO	24,327,973.29
23	30.84	0.31	8,11,14-Eicosatrienoic acid, (Z,Z,Z)-	779	306	C20H34O2	230,445.31
24	31.02	3.02	9-octadecenoic acid (Z)-	866	282	C18H34O2	2,248,164.28
25	32.01	1.10	8,11,14-eicosatrienoic acid,(Z,Z,Z)-	750	306	C20H34O2	816,400.44
26	33.19	1.31	9,12,15-Octadecatrienoic acid,2-(acetyloxy)-1-[(acetyloxy)methyl]ethyl ester, (Z,Z,Z)-	828	436	C25H40O6	974,799.45
27	36.46	0.62	Hexadecadienoic acid, methyl ester	742	266	C17H30O2	463,711.17
28	36.76	1.07	Arachidonic amide, N-[5-hydroxy-n-pentyl]-	745	389	C25H43NO2	792,734.00
29	36.90	0.91	7-methyl-Z-tetradecen-1-OL acetate	713	268	C17H32O2	673,210.27
30	37.23	0.84	HI-oleic safflower oil	742	450	C21H22O11	623,697.87
31	38.14	1.36	Panaxjapyne A	756	246	C17H26O	1,015,368.65
32	39.87	0.51	HI-oleic safflower oil	761	450	C21H22O11	382,042.69
33	40.08	0.47	1-heptatriacotanol	789	536	C37H76O	352,338.72

### 3.2 HPLC investigation of fermented MOLs phenolic and flavonoid compounds

A whole of 11 components containing phenolic acids and flavonoids were detected and measured by using HPLC in the fermented MOLs ethanolic extract. The main flavonoids and phenolic acids were determined at 425 nm (Table 4, Figures 1B, C). The primary detected phenolic acids were P-Coumaric acid (14.63 μg/mL), caffeic acid (9.42 μg/mL), gallic acid (4.66 μg/mL), ferulic acid (11.63 μg/mL), and catechin (8.62 μg/mL). The significant flavonoids detected by HPLC were naringenin (12.66 μg/mL), rutin (8.52 μg/mL), quercetin (7.45 μg/mL), kaempferol (6.78 μg/mL), luteolin (8.56 μg/mL), and apigenin (7.41 μg/mL).

**Table 4:** List of phytochemical components of fermented *Moringa oleifera* leaves used in the current study and identified by HPLC analysis.

**Peak no**.	**Name**	**Retention time**	**Height (mAU)**	**Area (mAU.s)**	**Concentration; Conc. (μg/mL)**
1	Naringenin	4.6	202.102	215.68	12.66
2	Rutin	5.2	500.410	330.85	8.52
3	Quercetin	7.0	1,201.011	448.52	7.45
4	Kaempferol	8.1	180.001	204.44	6.78
5	Luteolin	9.0	190.120	380.78	8.56
6	Apigenin	10.0	177.030	189.58	7.41
7	Catechin	12.01	195.255	320.09	8.62
8	P-Coumaric	6.0	1,300.051	450.12	14.63
9	Caffeic	8.0	700.420	320.70	9.42
10	Gallic	10.0	490.320	201.33	4.66
11	Ferulic acid	11.0	1,020.410	410.32	11.63

### 3.3 Clinical observations and kaplan-meier survival analysis

**Figure 2:**
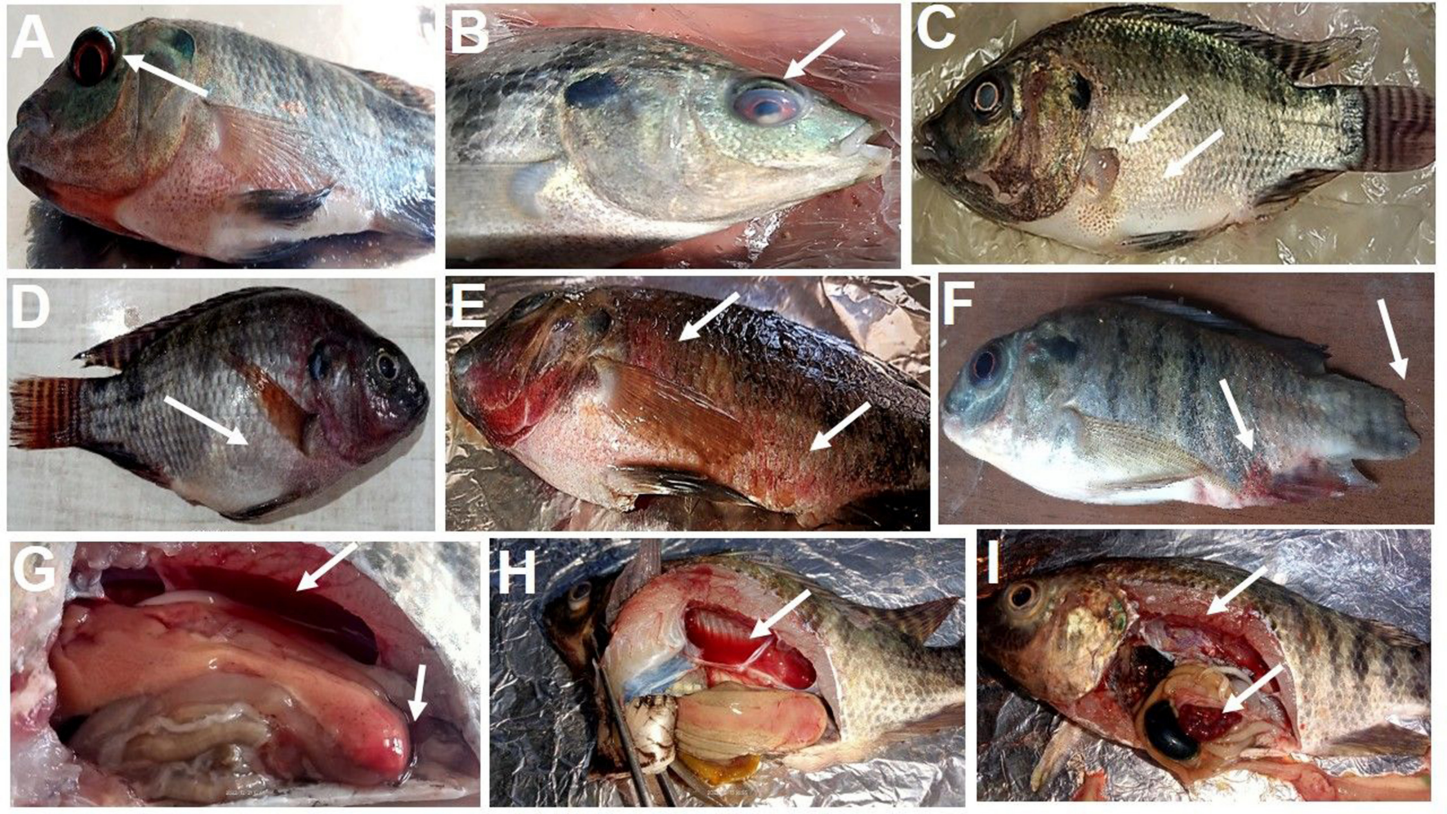
Clinical signs observed in tilapia during experimental infection by *A. hydrophila*. **(A)** exophthalmos, **(B)** corneal opacity, **(C)** fin rot, abdominal distention, **(D)** abdominal distention, ascites, **(E)** hemorrhagic septicemia, **(F)** fin erosion and hemorrhagic septicemia at the base of the fins and the anus, **(G)** enlarged yellowish and friable liver with hyperemic intestine filled with yellowish watery fluid, **(H)** hemorrhagic fluid in the abdominal cavity with enlarged yellowish friable liver, **(I)** enlarged gall bladder, congestion of all internal organs.

As shown in Figure 2. The positively infected Nile tilapia showed classic symptoms of *A. hydrophila* infection between 24 and 120 h post-challenge. Signs included hemorrhagic septicemia, exophthalmia, corneal opacity, fin erosions, ascites, and hemorrhagic congestion of internal organs. Fish moderately exhibited clinical signs in the MOLP-supplemented groups (MOLP at 50 and 100 g/kg diet). At the same time, few observed clinical signs were exhibited by fish in the MOLF-supplemented groups (MOLF at 50 and 100 g/kg diet) compared with the positive C group (challenged with *A. hydrophila*), which exhibited severe clinical signs and postmortem lesions. Data is shown in Table 5.

**Table 5:** RPS of Nile tilapia infected with *A. hydrophila* and fed a diet supplemented with different doses of dried or fermented *Moringa oleifera* leaves.

**Groups**	**C +ve**	**MOLP-L**	**MOLP-H**	**MOLF-L**	**MOLF-H**	**C-ve**
Number of dead animals	10 (15)	7 (15)	5 (15)	4 (15)	2 (15)	-
Mortality (%)	66.70%	46.67%	33.30%	26.67%	13.33%	-
Survival (%)	33.30	53.33	66.70	73.33	86.66	-
RPS^a^	-	30.03	50.07	60.1	80.1	-
Hemorrhagic septicemia	2.60 ± 0.13^a^	1.93 ± 0.06^b^	1.67 ± 0.12^bc^	1.40 ± 0.13^c^	0.53 ± 0.13^d^	0.067 ± 0.06^e^
Exophthalmia	2.67 ± 0.12^a^	2.06 ± 0.11^b^	1.40 ± 0.13^c^	1.20 ± 0.17^cd^	0.73 ± 0.15^d^	0.00 ± 0.00^e^
Corneal opacity	2.73 ± 0.11^a^	2.20 ± 0.10^ab^	2.13 ± 0.16^b^	1.40 ± 0.13^c^	1.13 ± 0.16^c^	0.00 ± 0.00^d^
Fin-erosions	2.60 ± 0.13^a^	2.06 ± 0.06^ab^	1.93 ± 0.11^b^	1.53 ± 0.13^b^	0.80 ± 0.17^c^	0.067 ± 0.06^d^
Ascites	2.73 ± 0.12^a^	2.13 ± 0.35^b^	1.93 ± 0.15^bc^	1.60 ± 0.13^c^	0.80 ± 0.14^d^	0.06 ± 0.06^e^
Hemorrhagic congestion of internal organs	2.73 ± 0.11^a^	2.00 ± 0.09^bc^	1.86 ± 0.09^bc^	1.73 ± 0.11^c^	0.87 ± 0.13^d^	0.06 ± 0.06^e^

^a^Relative percentage of survival (RPS), calculated by the equation: RPS = [1- (% mortality of treated group, % mortality of control group)] × 100. Clinical signs and autopsy lesions were estimated by semi-quantitative methods as follows: “0 = absence of lesion, 1 = mild, 2= moderate, and 3 = severe lesions”. Examined fishes = 15 fishes/group. There was no significant difference between means carrying the same alphabetical superscript letter in the same row (^*^P ≤ 0.05).

**Figure 3:**
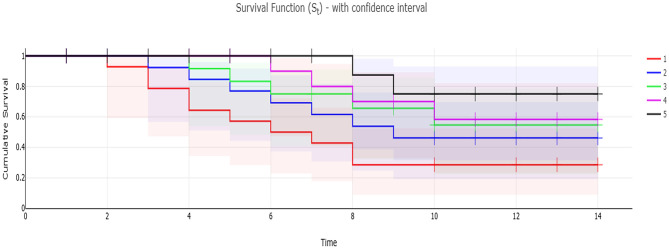
Kaplan-Meier survival analysis of Nile tilapia fed dietary supplementation of −ve control group, MOLP-H, MOLF-L, MOLP-H, and MOLP-L for 30 days and then challenged with *A. hydrophila* for 14 days. Data are shown as the mean ± SEM (*n* = 15 fish/group, *P* < 0.05). Line –Survival rate (St) per time. Confidence interval—added the Confidence interval areas to the line, with confidence level of (1 – α). S.E area—present St ± 1*S.E, the S.E is the area below and above the line. S.E error bars—present St ± 1*S.E, the S.E is presented as error bar. 1, −ve control group; 2, MOLP-L; 3, MOLP-H; 4, MOLF-L; 5, MOLF-H.

The obtained results of the Kaplan-Meier survival analysis are shown in Figure 3, are displayed that Nile tilapia fed a diet supplemented with MOLF (50 and 100 g/kg diet) displayed considerably reduced mortalities (*P* < 0.05) than those of the *C* tilapia when challenged with *A. hydrophila*. Tilapia in the control set exhibited a mortality rate of 66.7% compared to the (46.67%, 33.3%. 26.67%, and 13.33%) in the MOLP-L, MOLP-H, MOLF-L, and MOLF-H groups respectively. Table 5 shows the RPS of fish challenged with *A. hydrophila* and fed MOLP or MOLF-supplemented diets at (50 and 100 g/kg diet). There were dose-related effects with RPS of 30.00, 50.00, 60.00, and 80.00% of the groups fed diets supplemented with MOLP-L, MOLP-H, MOLF-L, and MOLF-H (50 and 100 mg/kg diet) when compared to control Nile tilapia.

### 3.4 Effect of MOLF or MOLP on immune and phagocytosis assay of Nile tilapia

**Figure 4:**
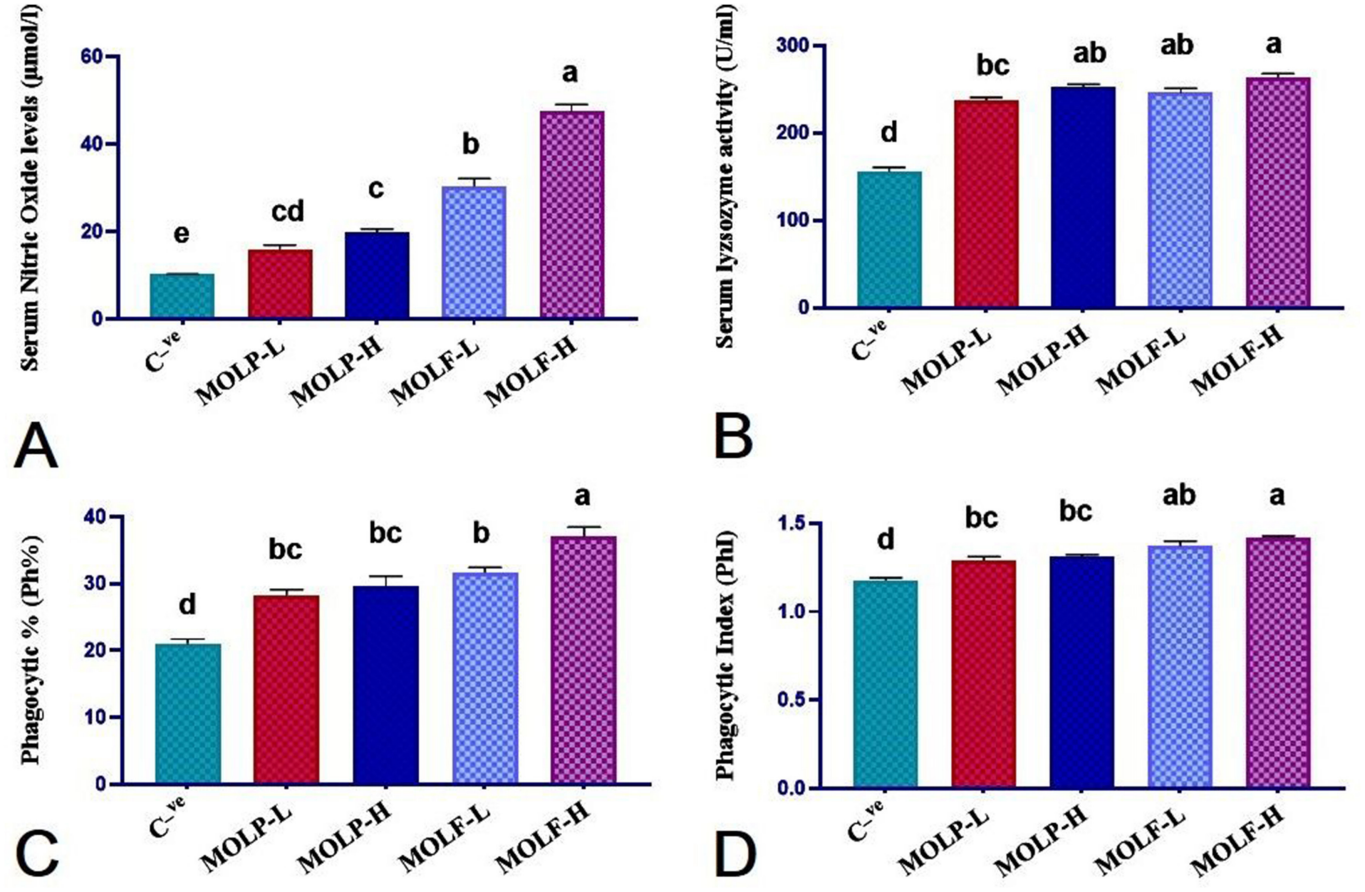
Effect of dietary supplementation of MOLF-H, MOLF-L, MOLP-H, and MOLP-L for 30 days on **(A)** Serum nitric oxide level (μmol/L), **(B)** Serum lysozyme activity (unit/mL), **(C)** Phagocytic percent (%), **(D)** Phagocytic index of Nile tilapia (*O. niloticus*). Data are shown as the mean ± SEM. *n* = 6 samples/group. Bars with different letters are significantly different at (*P* < 0.05).

The obtained results of NO and LYZ serum levels, phagocytic activities, and PhI of fishes fed MOLP or MOLF for a 30-day experimental period are shown in Figure 4. Significantly (*P* < 0.05), Nile tilapia*-*fed high-and low-fermented MOLs showed substantially higher NO serum levels (47.53 ± 1.59 and 30.44 ± 1.72) in the MOLF-H and MOLF-L, respectively, when matched with tilapia fed high-and low-dried MOLs (19.91 ± 0.70 and 15.95 ± 1.03), respectively, in the MOLP-H and MOLP-L, which were all markedly (*P* < 0.05) higher than the C group (10.26 ± 0.06). Nile tilapia fed high-and low-fermented MOLs and high and low-dried MOLs showed significantly (*P* < 0.05) higher LYZ serum levels (263 ± 4.93, 252.66 ± 3.1, 246 ± 5.01, and 237.83 ± 2.67) in the MOLF-H, MOLF-L, MOLP-H, and MOLP-L groups, correspondingly, when matched with the *C* tilapia (156 ± 4.64). The variances in the serum LYZ levels within tilapia sets fed MOLP-H, MOLP-L, MOLF-H, and MOLF-L were non-significant.

Nile tilapia fed high and low fermented MOLs and high and low dried MOLs showed significantly (*P* < 0.05) higher phagocytic % (37.00 ± 1.46, 31.66 ± 0.76, 29.66 ± 1.47 and 28.33 ± 0.76) in the MOLF-H, MOLF-L, MOLP-H, and MOLP-L groups, respectively when matched with the *C* tilapia (21.00 ± 0.73). The variances in the phagocytic % within tilapia sets fed MOLP-H, MOLP-L, and MOLF-L were non-significant (*P* < 0.217). Only the tilapia-fed MOLF-H was significantly (*P* < 0.0.05) higher in phagocytic % than other supplemented groups.

Nile tilapia fed high and low fermented MOLs and high and low dried MOLs showed significantly (*P* < 0.05) higher PhI (1.42 ± 0.009, 1.37 ± 0.029, 1.31 ± 0.013, and 1.29 ± 0.023) in the MOLF-H, MOLF-L, MOLP-H, and MOLP-L groups, respectively, when matched with the *C* tilapia (1.176 ± 0.012). The variances in the PhI between tilapia sets fed MOLP-H, MOLP-L, MOLF-H, and MOLF-L were non-significant.

### 3.5 Effect of MOLF or MOLP on hepatic oxidative stress biomarkers of Nile tilapia

**Figure 5:**
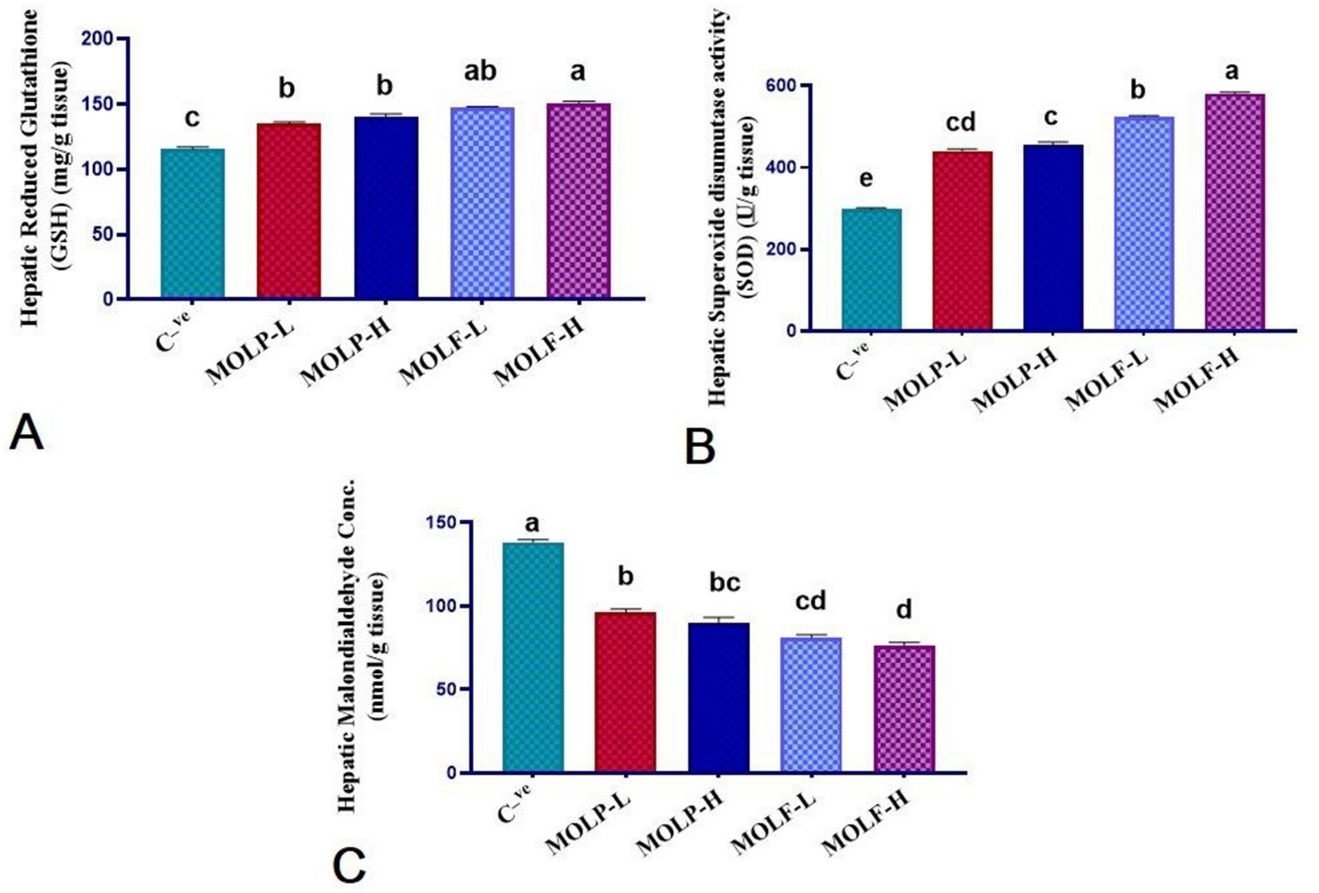
Effect of dietary supplementation of MOLF-H, MOLF-L, MOLP-H, and MOLP-L for 30 days on **(A)** reduced glutathione (GSH) (mg/g tissue), **(B)** superoxide dismutase (SOD) activity (*u*/g tissue), **(C)** malondialdehyde (MDA) (nmol/g tissue) of Nile tilapia (*O. niloticus*). Data are shown as the mean ± SEM. *n* = 6 samples/group. Bars with different letters are significantly different at (*P* < 0.05).

As presented in Figure 5, a marked (*P* < 0.05) increment of the GSH level was evident in the liver tissues of Nile tilapia fed high-and low-fermented and high-and low-dried MOLs-supplemented diets (150.30 ± 1.92, 147.36 ± 0.95, 140.70 ± 1.84, and 135.18 ± 1.30) in the MOLF-H, MOLF-L, MOLP-H, and MOLP-L groups, correspondingly, when matched with the *C* tilapia (115.46 ± 1.59). Supplementation of MOLF markedly (*P* < 0.05) elevated the GSH content more than the MOLP. Similarly, a marked (*P* < 0.05) increment of the SOD activities was evident in the liver tissues of Nile tilapia fed high-and low-fermented and high-and low-dried MOLs-supplemented diets (580.66 ± 3.1, 524 ± 3.28, 455.3 ± 6.75, and 440 ± 4.74), respectively, in the MOLF-H, MOLF-L, MOLP-H, and MOLP-L groups when matched with the *C* tilapia (297.66 ± 3.74). Supplementation of MOLF significantly (*P* < 0.05) elevated the GSH content and SOD activities more than the MOLP. Meanwhile, a marked decline of MDA concentration (*P* < 0.05) was evident in the liver tissues of Nile tilapia fed high and low fermented and high and low dried MOLs-supplemented diets (76.00 ± 2.19, 81.00 ± 1.82, 90 ± 3.28, and 96.00 ± 2.19) in the MOLF-H, MOLF-L, MOLP-H, and MOLP-L groups, correspondingly, when matched with the C group (138.33 ± 1.47). Supplementation of MOLF significantly (*P* < 0.05) reduced the MDA concentration compared to the MOLP.

### 3.6 Effect of MOLF or MOLP on the mRNA expression of immune-linked genes in the spleen and head kidney of Nile tilapia

**Figure 6:**
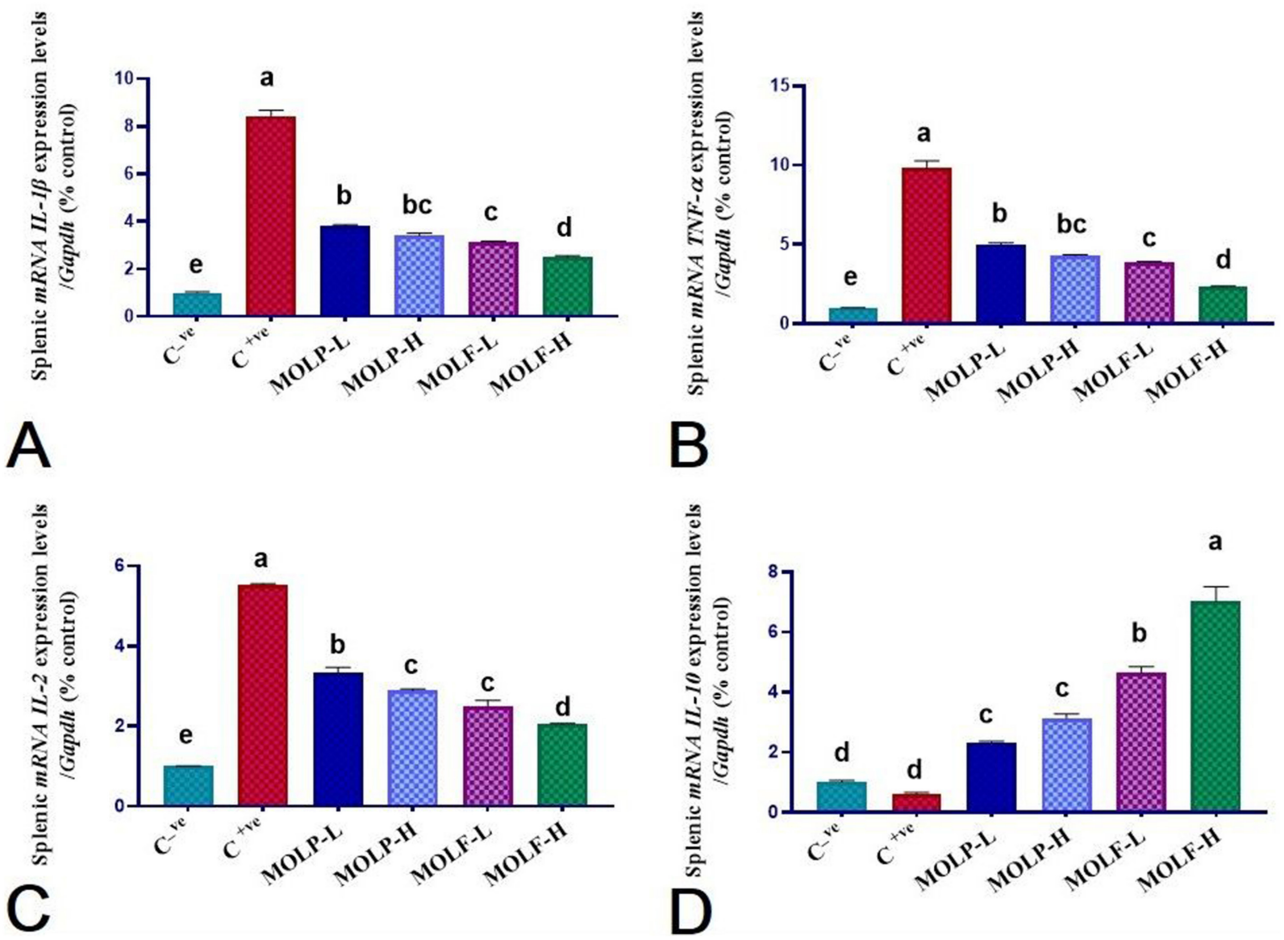
Effect of dietary supplementation of MOLF-H, MOLF-L, MOLP-H, and MOLP-L for 30 days on relative m RNA expression of **(A)** Spleen *IL-1*β, **(B)** Spleen *TNF-*α, **(C)** Spleen IL-2 and **(D)** Spleen *IL-10* of Nile tilapia (*O. niloticus*). Data are shown as the mean ± SEM. n=3 samples/group. Bars with different letters are significantly different at (*P* < 0.05).

**Figure 7:**
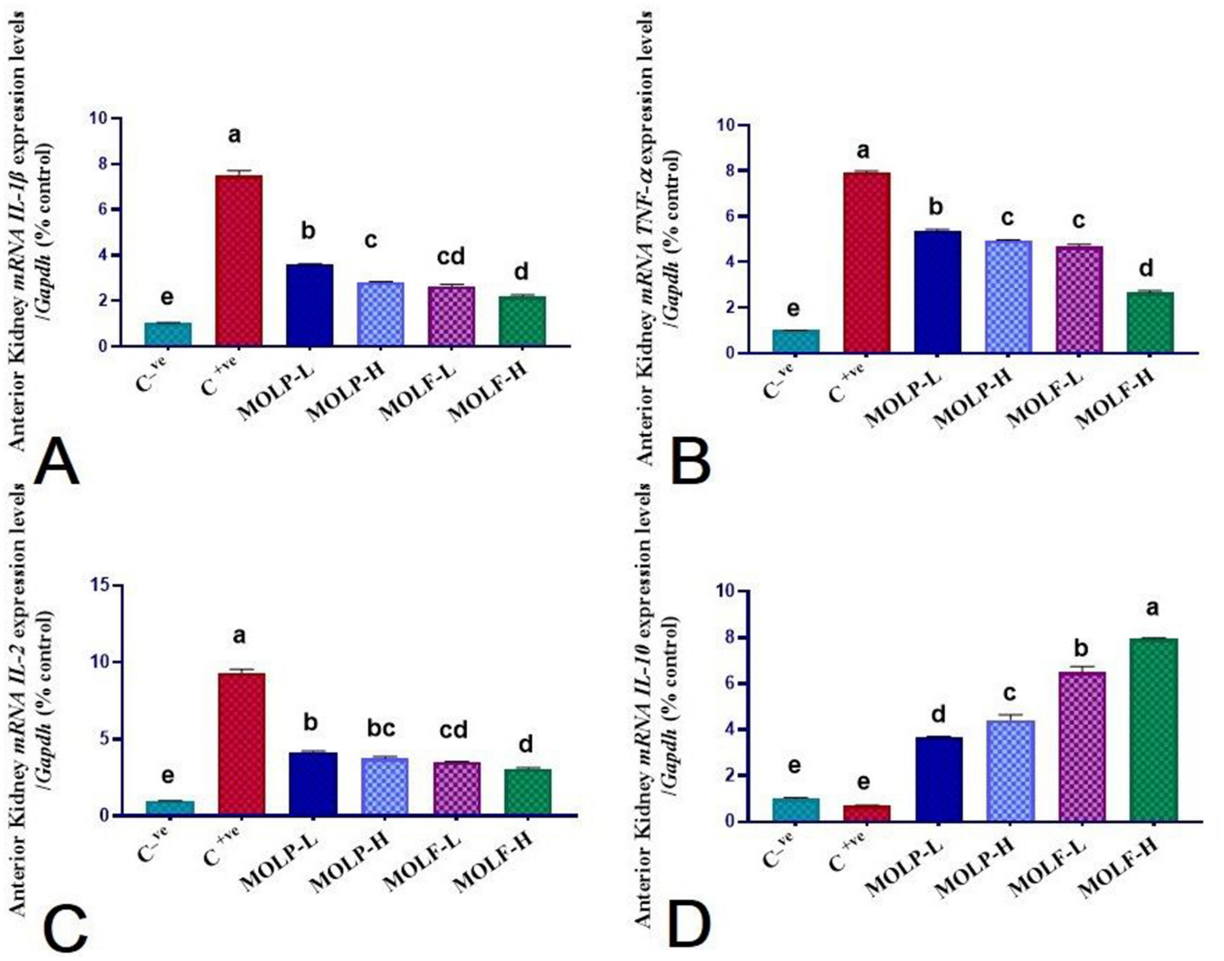
Effect of dietary supplementation of MOLF-H, MOLF-L, MOLP-H, and MOLP-L for 30 days on relative m RNA expression of **(A)** Anterior kidney *IL-1*β, **(B)** Anterior kidney *TNF-*α, **(C)** Anterior kidney *IL-2*, and **(D)** Anterior kidney *IL-10* of Nile tilapia (*O. niloticus*). Data are shown as the mean±SEM. *n* = 3 samples/group. Bars with different letters are significantly different at (*P* < 0.05).

As shown in Figures 6, 7, immune functioning-related genes, including *ILL-*β, *TNF-*α, *and IL-2*, were significantly (*P* < 0.05) upregulated; meanwhile, anti-inflammatory gene *IL-10* was significantly (*P* < 0.05) downregulated in the spleen and head of the kidney of Nile tilapia positive C group (+ve Control infected with *A. hydrophila* without any diet supplements) compared with the negative C group (non-infected Nile tilapia). On the contrary, a substantial (*P* < 0.05) correction of the analyzed genes was verified in the Nile tilapia-fed diets supplemented with MOLF and MOLP at high and low doses compared with the positive control group. Of note, notably, Tilapia-fed MOLF-supplemented diets exhibited a marked (*P* < 0.05) restoration in the mRNA regulation of the analyzed genes in the spleen and anterior kidney compared to the tilapia-fed MOLP-supplemented diets.

### 3.7 Effect of MOLF or MOLP supplements on the bacterial load (CFU) in the spleen and liver of Nile tilapia

**Figure 8:**
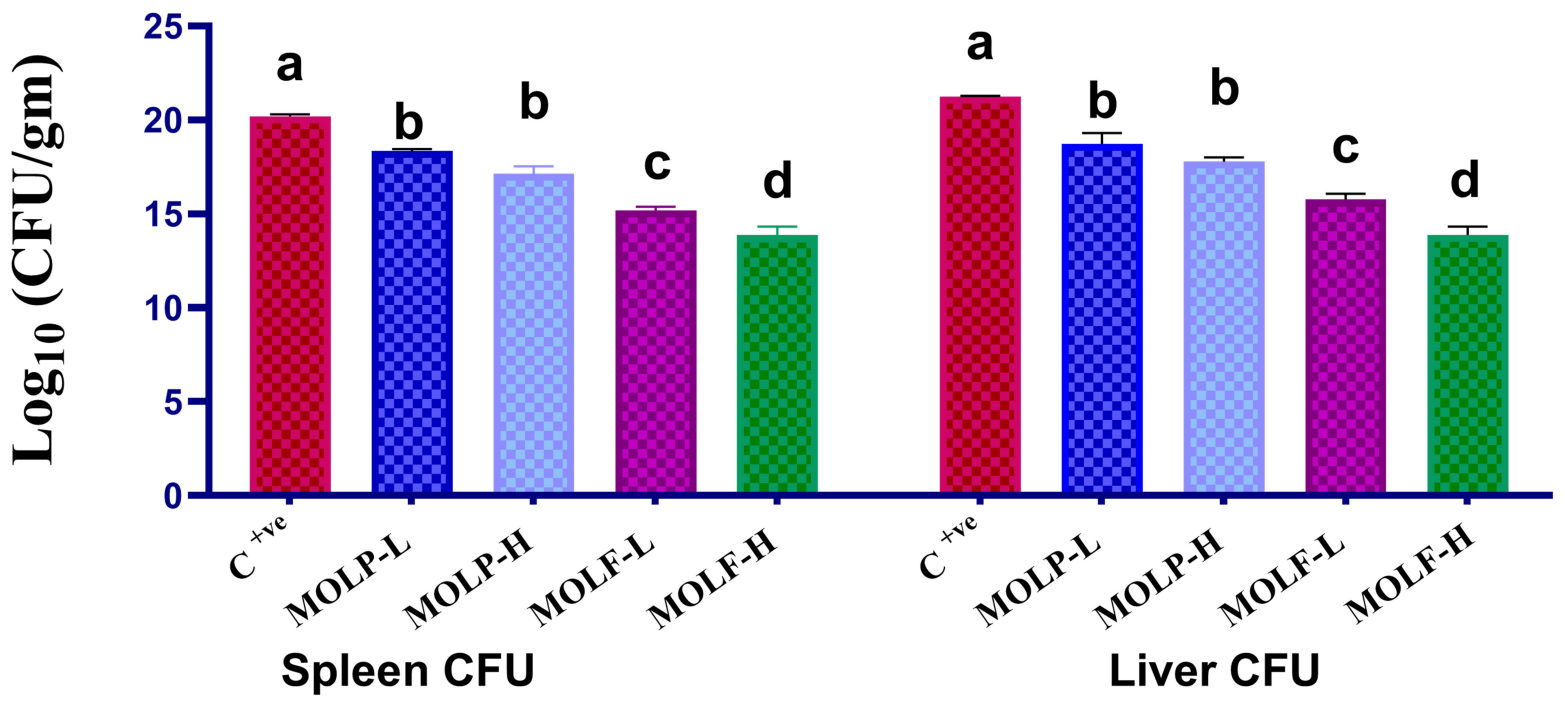
Effect of dietary supplementation of MOLF-H, MOLF-L, MOLP-H, and MOLP-L for 30 days on bacteriological colony count (CFU/g) of the challenged Nile tilapia fish (*O. niloticus*) spleen and liver. Data are shown as the mean ± SEM. *n* = 3 samples/group for each organ. Bars with different letters are significantly different at (*P* < 0.05).

The data obtained declared that dietary supplements of fermented and dried MOLs to Nile tilapia significantly decreased the total bacterial count CFU of the *A. hydrophila* in the spleen and liver tissues compared to those of the *C* tilapia. Data of the *A. hydrophila* CFU per g tissue among all groups is shown in Figure 8. A significant (*P* < 0.05) decline of the *A. hydrophila* CFU was evident in the spleen tissues of Nile tilapia that received high-and low-fermented and high-and low-dried MOLs-supplemented diets (13.88 ± 0.45, 15.18 ± 0.21, 17.13 ± 0.41, and 18.34 ± 0.11) respectively, in the MOLF-H, MOLF-L, MOLP-H, and MOLP-L groups compared with the positive *C* tilapia (infected with *A. hydrophila*) (20.19 ± 0.11). Supplementation of MOLF considerably (*P* < 0.05) declined the *A. hydrophila* CFU in the spleen more than the MOLP. Similarly, a significant (*P* < 0.05) decline of the *A. hydrophila* CFU was evident in the liver tissues of Nile tilapia fed high-and low-fermented and high-and low-dried MOLs-supplemented diets (13.89 ± 0.76, 15.75 ± 0.32, 17.78 ± 0.22, and 18.72 ± 0.58), respectively, in the MOLF-H, MOLF-L, MOLP-H, and MOLP-L groups than the positive *C* tilapia group (infected with *A. hydrophila*) (21.24 ± 0.043). Supplementation of MOLF considerably (*P* < 0.05) declined the *A. hydrophila* CFU in the liver more than the MOLP.

## 4 Discussion

Using dietary nutrient additives as novel instruments for improving immune functioning and lowering disease incidences is considered safe, non-toxic, economical, and environmentally beneficial. Several nutritional immunostimulants are utilized to increase aquaculture output (51). Immunostimulant feed supplements upregulate immune-linked genes besides enhancing cellular and humoral immune responses in blood and head-kidney (52), as well as the immune reactivity against infections in aquatic species (53). In this study, authors set out to investigate the effect of lactic fermentation in enhancing the nutritious and phytochemical characteristics of MOLs and innate immune activities of *O. niloticus*, thereby alleviating their tolerance vs. the *A. hydrophila* challenge.

Our obtained results of the MOLF HPLC analysis revealed a significantly high content of the phenolic and flavonoid components. Current results showed that L.plantarum fermentation enriched the polyphenols and flavonoid synthesis in MOLs through different enzymatic reactions (54). The HPLC analysis results of fermented *M. oleifera* leaves showed several flavonoids (naringenin, rutin, quercetin, kaempferol, luteolin, apigenin, and catechin) in addition to phenolic compounds (p-coumaric, caffeic, gallic, and ferulic acids) that were significantly amplified by fermentation. In addition, we detected that the fermented MOL has kaempferol, which could not be identified in the raw MOLP. Our results suggested that *L. plantarum* fermentation improved the biological properties of MOL by increasing phytochemical production. Many studies reported that catechin, ellagic, kaempferol, and quercetin may overcome inflammatory reactions (55, 56). Catechin displayed an essential anti-inflammatory role via regulating the ferroptosis pathway (57). Moreover, kaempferol, quercetin, and ellagic notably decreased inflammation using reduced expression of proinflammatory cytokines and production of NO (58, 59). Therefore, its higher polyphenol concentration may amplify MOLF's anti-inflammatory benefits. One possible use for *L. plantarum*-fermented MOLs is as an addition to feed. In comparison to MOLP, we propose that MOLF is more active.

Regarding the effect of fermented MOLs, the current study results exhibited that high and low levels of MOLF significantly enhanced NO and LYZ and the phagocytic efficacy and PhI of Nile tilapia. The obtained data are in agreement with (20, 60). MOLP at a level of 15% was more effective than 10% and 5% in enhancing serum LYZ activity in guppy fish (20). Similarly, incorporating MOLP into the diet of Nile tilapia at 5% levels was more effective than at 1.5% levels (13). The phenolic compounds detected in MOLs, including quercetin, kaempferol, flavonoids, and anthocyanins, are linked to enhanced NO levels (61). Furthermore, the phytochemical content of Moringa leaves, such as carotenoids, minerals, vitamins, moringinine, alkaloids, flavonoids, sterols, phytoestrogens, and caffeoylquinic acids, may contribute to these observed effects (38, 62).

Oxidative stress occurs when pro-oxidant and antioxidant levels are imbalanced. The resultant ROS can induce oxidative damage in macromolecules, including proteins, nucleic acids, and lipids. The present study revealed that GSH, SOD, and MDA were altered as biomarkers of oxidative damage. MOLs significantly increased GSH content and SOD activities but lessened MDA concentrations in the liver tissue of Nile tilapia-fed fermented MOLs more significantly than dried MOLs powder. These results are aligned with the previous report (60, 63). MOL's relative abundance of polyphenols (phenyl propanoids, phenolic acids, favonoids, and tannins) directly enhances the antioxidant response of O. niloticus by acting as hydrogen donors that stabilize free radicals generated by cells through electron donation or acceptance (64). The potent antioxidant activity of Moringa species is attributed to their abundant phenolic content, which functions as an antioxidant molecule by stabilizing free radicals produced within a cell by either receiving or giving electrons (65). The findings of the current study may be attributed to the presence of vitamin C, carotenoids, tocopherols, flavonoids, and other phenolic substances in M. oleifera (66). The presence of glucosinolates, which carry a benzyl-glycoside and undergo enzymatic hydrolysis to isothiocyanates, thiocyanates, or nitriles, further boosts the antioxidant activity in MOLs (67, 68).

Feeding *Megalobrama amblycephala* juveniles with 2.2% and 4.4% of FMOL for 8 weeks enhanced antioxidant activities in the liver and downregulated the transcriptional levels of inflammatory response-linked genes, including *NF-k*β and *TLR-4* thus enhancing hepatic anti-inflammatory response (63). Leaves of *M. oleifera* possess high contents of flavonoids and phenolic compounds, including quercetin, as well as high content of ascorbic acid and vitamin A. Dietary supplements with *M. oleifera* improved the antioxidant potentials of aquatic animals, including Nile tilapia (13, 69). Similarly, MOLF significantly increased the GSH content in the liver of tilapia fed on MOLF-H and MOLF-L. GSH scavenges ROS to relieve oxidative stress directly (70). The data obtained in this study revealed that MOLF enhanced the antioxidant capacity in the liver of *O. niloticus*, which was more significant in MOLF dietary supplements than in raw MOLP.

The immunological response of Nile tilapia to Aeromonas infections has been previously studied using several traditional immune indicators, such as leukocyte counts, red blood cell counts, LYZ (71), and immunoglobulin M (3). Nevertheless, the molecular mechanisms that mediate the early innate immune response remain largely unknown. Gene expression analysis is useful for examining host-pathogen interactions and locating crucial cellular functions during infection.

This knowledge can facilitate the creation of efficient control methods for bacterial infections in tilapia farming. The study employed a local strain of *A. hydrophila* to infect Nile tilapia, and within the first 120 h after the challenge, the proportional regulations of specific genes (*IL-1*β, *TNF-*α, *IL-2*, and *IL-10*) involved in early innate immunity reactions in the spleen and anterior kidney were examined. These genes could determine the critical elements required to successfully combat *A. hydrophila* infection, a leading cause of disease-related mortality outbreaks on Egyptian Tilapia farms, by describing and learning more about the complex dynamics of host-pathogen interactions during the early stages of infection. The outcomes of the current protocol exhibited that supplementation of MOLF meaningfully upregulated the *IL-1*β, *TNF-*α, and *IL-2* and downregulated the *IL-10* transcriptional expression levels in the spleen and anterior kidney of Nile tilapia post *A. hydrophila* infection. Parallel results were exhibited where the *INF-*γ*, IL-10*, and *TNF-*α genes were significantly upregulated in Nile tilapia spleen supplemented with MOLs (72). In contrast, the *IL-1*β gene regulation was markedly downregulated in the tilapia that received MOLF-and MOLP-supplemented diets.

The key factors for inflammatory responses are macrophages (73). Macrophages produce proinflammatory and inflammatory intermediaries, including *IL-1*β*, TNF-*α*, IL-6, NO, iNOS*, and *ROS*, prompting cellular injury (37). In the present study, MOLF significantly downregulated *IL-1*β, *TNF-*α, and *IL-2* expression in Nile tilapia” 'spleen and anterior kidney compared with the positively infected fish groups. Fermentation could increase anti-inflammatory potentials by transforming the phenolic combinations (36). MOLF showed high contents of catechin and quercetin, which have anti-inflammatory capabilities via modulation of *IL-1*β*, TNF-*α, and NO (74). So, such components may strengthen the anti-inflammatory effects of MOLF. Inflammation is associated with high cellular ROS levels that modulate the proinflammatory cytokines via transcriptional upregulation of inflammation-associated genes (75). Our results showed that *A. hydrophila* challenge induced oxidative stress, which can be markedly alleviated by MOLF dietary supplementation. Kaempferol is a powerful flavonoid that could decrease ROS production (76). In our study, kaempferol was in the MOLF-fermented L. plantarum. In contrast, the ameliorating effect of MOLF on oxidative stress may be attributed to the ' 'MOLF's kaempferol content.

A key modulator of the adaptive immune response to microbial infections, *IL-1*β is the initial proinflammatory cytokine produced. When a disease (or damage) occurs, *IL-1*β sets off a series of events that produce systemic and local reactions that result in inflammation, allowing the host to react to infections quickly. The increase or downregulation of the expression of additional effector molecules mediates the actions of *IL-1*β. These signaling pathways activate transcription factors, which cause the synthesis of cell adhesion molecules, chemokines, cytokines, and acute-phase proteins (77, 78). Following IP injection challenges with *A. hydrophila* and *Pseudomonas fluorescens*, the proportional *IL-1*β gene regulation outcomes in Nile tilapia in different organs were assessed. It was discovered that the infected fish had considerably higher levels of this gene than the uninfected controls (79).

Contrary to our results, fermented moringa significantly decreased *TNF-*α and *IL-1*β after *A. hydrophila* infection (60). They suggested that this was due to the ability of polysaccharides and flavonoids to regulate macrophage activity, normalizing the proinflammatory cytokine expression levels in gibel carp. Increased MOL levels in diets reduced the expression of *TNF-*α and *IL-1*β *mRNA* in the skin and gills of *S. aurata* (80). This decrease may be linked to MOLs' inhibitory effects on the phosphorylation of inhibitor *k*β protein and mitogen-activated protein kinases (81). Feeding Nile tilapia with 0.5% or 1% methanolic extract of *M. oleifera* seeds decreased the *TNF-*α and increased the *IL-1*β expression in the liver (82).

## 5 Conclusion

The combined results from the current work advocate that the nutritional supplement of *L. plantarum* fermented *M. oleifera* leaves to Nile tilapia feeds could enhance the immune-related responses, antioxidant potential, and mRNA regulation of immunity-related genes, along with enhancing Nile tilapia” 'resistance to *A. hydrophila* challenge. This might be mediated mainly via the acid fermentation process that increases the amounts of health-enhancing active components in leaves of *M. oleifera*, decreases toxicological antinutritional factors, and improves the antioxidant status. Notably, according to this study, fermented MOLF exhibited more significant potential for health benefits in the fight against infections than the raw MOLP, and concurrent supplementation of fermented *M. oleifera* leaves markedly exhibited anti-inflammatory potentials against the detrimental impacts of *A. hydrophila* infection of Nile tilapia. Further study of the immunostimulants effects of MOLF against immune-toxic chemicals in aquaculture as well as their neuroprotective potentials in long term studies are recommended.

## Data Availability

The original contributions presented in the study are included in the article/supplementary material, further inquiries can be directed to the corresponding authors.
